# Photographs of manipulable objects are named more quickly than the same objects depicted as line-drawings: Evidence that photographs engage embodiment more than line-drawings

**DOI:** 10.3389/fpsyg.2014.01187

**Published:** 2014-10-21

**Authors:** Joshua P. Salmon, Heath E. Matheson, Patricia A. McMullen

**Affiliations:** Department of Psychology and Neuroscience, Dalhousie UniversityHalifax, NS, Canada

**Keywords:** manipulability, action, naming, photographs, line drawings, perception, identification, embodied cognition

## Abstract

Previous research has shown that photographs of manipulable objects (i.e., those that can be grasped for use with one hand) are named more quickly than non-manipulable objects when they have been matched for object familiarity and age of acquisition. The current study tested the hypothesis that the amount of visual detail present in object depictions moderates these “manipulability” effects on object naming. The same objects were presented as photographs and line-drawings during a speeded naming task. Forty-six participants named 222 objects depicted in both formats. A significant object depiction (photographs versus line drawing) by manipulability interaction confirmed our hypothesis that manipulable objects are identified more quickly when shown as photographs; whereas, non-manipulable objects are identified equally quickly when shown as photographs versus line-drawings. These results indicate that factors such as surface detail and texture moderate the role of “action” and/or “manipulability” effects during object identification tasks, and suggest that photographs of manipulable objects are associated with more embodied representations of those objects than when they are depicted as line-drawings.

## INTRODUCTION

The theory of embodied cognition has increasingly gained support in explaining much of human cognition over the last decade (for a recent review, see Borghi and Pecher, 2011). Though there is (as of yet) no unified model of theory of embodied cognition, most accounts suggest that “cognitive activity is grounded in sensory-motor processes and situated in specific contexts and situations” (Borghi and Pecher, 2011, p. 1). A prediction of this theory is that the same neural substrate activated when perceiving an object is also used to represent or form a memory of the object. For example, to talk about a “hammer,” one activates the same neural networks that are active when actually seeing and using a hammer. This raises interesting questions about the differences in representations between objects we regularly use and pick up (i.e., manipulable objects) versus those that we do not functionally use (i.e., “non-manipulable” objects). Specifically, if manipulable objects are defined in part by learned motor-associations, then these objects will activate motor-areas as a part of their object representation in a way that won’t be observed for non-manipulable objects. There is abundant neuropsychological (e.g., Warrington and Shallice, 1984) imaging (see Thompson-Schill, 2003), and behavioral (e.g., Masson et al., 2011; see also Barsalou, 2008) evidence that this is the case. Importantly, we have recently shown that photographs of manipulable objects (i.e., those that can be grasped for use with one hand) are named more quickly, but categorized at a higher level more slowly, than non-manipulable objects (when matched for object familiarity and age of acquisition (AoA); Salmon et al., 2014; see also Filliter et al., 2005). We interpreted this “manipulability effect” as evidence for differential access of manipulable versus non-manipulable object representations due to involvement of distributed motor associations. Importantly, in that study, photographs of objects were used. In the current research we investigate whether the type of depiction of the object influences the extent to which manipulability effects are observed. Specifically, line-drawings depict a more simplified and basic-features version of the object, and are generally reduced in the intensity and quality of surface details, texture, shading, and 3-D visual cues. In the present experiment we investigate whether line-drawings activate embodied object representations to the same degree as more realistic photographic depictions.

The Snodgrass and Vanderwart (1980) standardized set of line-drawings has been commonly used for many studies of object recognition. It is assumed that processing line-drawings is equivalent to processing more realistic depictions of the objects, such as photographs or 3-D, real objects. However, this assumption may be invalid for certain kinds of behaviors (e.g., Price and Humphreys, 1989; Nicholson and Humphrey, 2001). In particular, manipulable objects, defined as those objects we can pick up and use with one hand, have certain visual properties that indicate manipulability. In the case of tools this could simply be the presence of a handle; in the case of fruits and vegetables some kind of roundness or elongation that affords^1^ a place to grasp the object. If detecting these properties (i.e., smoothness and texture) is dependent on the amount of surface detail present, a photograph of an object should be more readily identified as manipulable, than a less detailed line drawing of the same object.

Most object-recognition research in the past has focused on the role of color as opposed to surface detail of objects (Brodie et al., 1991; Wurm et al., 1993; Tanaka and Presnell, 1999; Laws and Hunter, 2006; Therriault et al., 2009). However, recent evidence has suggested independent effects of form, color, and texture/surface detail during speeded-classification of objects (Cant et al., 2008), as well as separate ventral-stream brain areas for form, color, and texture (Cant and Goodale, 2007; Cavina-Pratesi et al., 2010).

Specific evidence that the quality of object depiction is important comes in part from research showing that object naming is facilitated by both congruent surface color (i.e., objects shown in colors typically found such as an orange) and photographic detail, although the effects of each combine under-additively (cf. Price and Humphreys, 1989). For instance, Nicholson and Humphrey (2001) reported that additional surface cues to object depictions reduced the latency to name rotated objects, indicating the importance of surface cues during an object-naming task. Additionally, Rossion and Pourtois (2004) found that the addition of texture and shading (without color) slightly improved naming agreement scores for the objects, but the effect of color was stronger, with the addition of color information unambiguously improving naming accuracy, and speeded correct responses times.

In the present behavioral experiment, we investigated the idea that if the object-action system and identification systems are integrated in an embodied way, one would expect faster identification of manipulable objects when more realistic depictions (i.e., surface detail) are available. For line-drawings, information indicating manipulability would be less readily available and hence manipulable objects would lose their processing advantage during identification. Critically this investigation was done as a within-subjects design with each participant naming the line-drawing and photographic versions of the same objects, and so serving as their own control. We hypothesized that manipulable objects would be identified more quickly when presented as photographs than as line drawings, given that surface details are a particularly important part of visually parsing out a 3-D manipulable object. This effect would appear as a two-way interaction between object depiction (line-drawing versus photograph) and object manipulability (manipulable versus non-manipulable objects) during object naming.

## MATERIALS AND METHODS

### PARTICIPANTS

Forty-six participants (6 males, 17–25 years, Mage = 19.67 years) enrolled as students at Dalhousie University, Halifax, N. S. volunteered to participate in this experiment. Seven were left-handed, and all had English as a first-language, normal, or corrected-to-normal vision, and no known history of neurological or visual disorders. This research was approved by the Dalhousie University Research Ethics Board.

### MATERIALS

**FIGURE 1:**
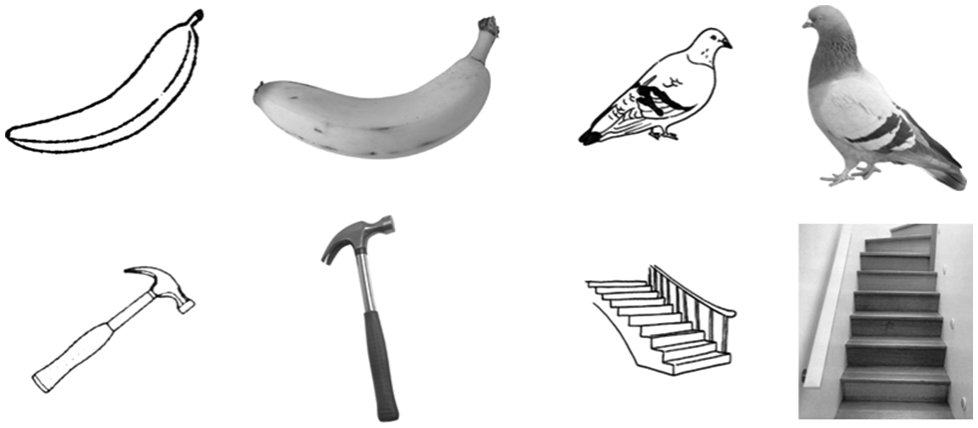
**Examples of photographs and line drawings of the same objects, with “manipulable” objects in the left-most columns, and “non-manipulable” objects in the right-most columns**.

Black and white photographs from the stimulus set normed by Salmon et al. (2010) were chosen on the basis that line-drawing depictions of the same objects were available from the Snodgrass and Vanderwart (1980) set. To further increase the number of objects available, a local artist (Andrea Rankin, http://andrearankin.tumblr.com/) was hired to draw 27 additional line-drawings in a style similar to that of Snodgrass and Vanderwart (1980). The result was a total of 222 objects that were depicted as both line-drawings and photographs. Examples of what the objects looked like are included in **Figure 1**, for a complete list of the items used, and examples of these new line drawings, please refer to the supplementary materials.

All stimuli were shown using the software DirectRT. Photographs were 4–11 cm tall and wide with visual angles of 4.3°–11.7°. The line-drawings, on the other hand, appeared slightly smaller on the screen (2–9 cm) subtending visual angles between 2.2 and 9.6° in both directions. It could be argued that this might give the line-drawings a slight disadvantage (smaller object depictions) or a slight advantage (more of object presented at fixation). However, *post hoc* analyses indicated that there was no significant correlation between the size of the image as it appeared on the screen, and the speed at which it was identified, *r*(444) = 0.07, *p* = 0.154. Furthermore, it was the interaction between object depiction and manipulability that was of key interest, not the main effect of object depiction.

### METHODS

All participants named both the line-drawing and photographic versions of each object. Object depiction was a blocked variable such that half of the participants named the photographs first and the other half named the line drawings first (i.e., counter-balanced). Prior to each block of object depiction (line drawings or photographs), participants were provided with a practice block (12 objects) of objects depicted in the same way as the block (i.e., line drawing or photograph, respectively). For example, a participant would practice with line-drawings, then complete the full line-drawing block, then practice with photographs, followed by the full photographs block. Participants were not told that the second block would contain the same items presented in the first.

Each trial was preceded by a fixation point that was displayed for 1 s. Each stimulus remained on the screen until it was named. The experimenter was in the room throughout all stimulus presentations and coded errors using a keyboard with an extension cord that saved the data directly to the computer used for presentation. The entire experiment took less than 1 h to complete.

### DATA ANALYSIS

#### A note on analysis of variance (ANOVA) versus mixed-effects analysis

Traditionally, reaction time (RT) data from object recognition tasks have been analyzed with analyses of variance (ANOVA; cf. Filliter et al., 2005; Wolk et al., 2005; Kalénine and Bonthoux, 2008 Kalénine et al., 2009). These analyses require aggregating the data prior to conducting the analysis. This aggregation process requires collapsing RT data either across subjects/participants or items. With each analysis, significant findings can then presumably be generalized to other subjects and items. In cases where it is useful to generalize across both subjects and items, both analyses are conducted (i.e., a separate subject, and a separate items analysis). Ideally, both analyses converge on the same results.

A growing trend in the analysis of RT data has been to use linear mixed-effects models (Agresti, 2002) instead of the traditional ANOVA analysis (cf. Kliegl et al., 2010; Lawrence and Klein, 2012). This analysis is often conducted using the lme4 package (Bates, 2007) in R (www.r-project.org), and, unlike the ANOVA, does not require aggregating data. This new method for analysis of linguistic data has been advocated by other linguistic researchers (e.g., Baayen, 2008; Kliegl et al., 2010) and researchers interested in stimulus-driven effects (e.g., Malcolm et al., 2008; Lawrence and Klein, 2012). The mixed-effects regression model has a number of advantages over standard repeated-measures ANOVA. First, a mixed-effects model can account for the effects of subjects and items at the *same time*. Second, the mixed-effects model can analyze the effect of manipulability as a continuous variable (instead of a categorical variable as is the case with the ANOVA). Finally, because there is not aggregation, mixed effects models have more statistical power than traditional analyses based on the ANOVA (Baayen, 2008).

#### Linear mixed effects analysis

Data analysis consisted of a mixed-effects analysis (conducted in R), followed by a more traditional repeated measures ANOVA subjects analysis (in SPSS) to confirm that the more traditional analysis supported the findings from the mixed-effects analysis. Analyses were conducted on RTs from correct response trials only (92% of the trials), with outlier rejection for RTs over 3 s, and less than 300 ms (0.4 % of the remaining trials). The key variables of interest from the mixed-effects model were Block (block 1 or block2), Manipulability (Manip), Depiction (photographs or line drawing), and the interaction between Manipulability and Depiction. Importantly, Manipulability was based on the first type of Manipulability ratings collected by Salmon et al. (2010), defined as the “extent to which an object can be grasped and used with one hand”; average ratings were used to treat Manipulability as a continuous variable in this analysis with scores from 1 (low manipulability) to 5 (high manipulability).

Covariate variables of AoA and familiarity (Fam) were also entered into the model to ensure that significant effects were not attributable to these covariates. Again, these were treated as continuous variables, with mean values taken from Salmon et al. (2010). AoA was defined as “the age at which you likely learned the name of the object” and ranged from 1 (acquired early) to 7 (acquired late). Fam was defined as “familiarity with the object” or “degree to which raters came into contact with or thought about the concept on a day-to-day basis,” and ranged from 1 (low familiarity) to 5 (high familiarity; Salmon et al., 2010, pp. 84–85).

To conduct the linear mixed effects analysis, both the subject error and item error accounted for by each trial were entered into the model.

$$\begin{array}{c} \mathrm{Pr}(yRT)=b_{0}+b_{1}\times {Block}_{si}+b_{2}\times {AoA}_{si}+b_{3}\times {Fam}_{si}+b_{4}\times {Depiction}_{si}+b_{5}\times {Manip}_{si}+b_{6}\times \\ ({Depiction}_{si}\times {Manip}_{si})+{subject}_{s}+{item}_{i}+\varepsilon_{si} \end{array}$$

where Pr(y_RT_) is the “probability of obtaining a given RT.” The variables b_0_, b_1_, b_2_, b_3_, b_4_, b_5_, b_6_ were the fixed effects coefficients, i.e., the group average effects induced by the variables Block_si,_ AoA_si,_ Fam_si,_ Depiction_si,_ Manip_si_, and the Depiction^∗^Manip_si_ interaction term. The random effect term subject_s_ represented each subject’s deviation from the group RT average. Likewise, the random effect item_i_ represented each items deviation from the item group RT average. Finally, ε_si_ represented the residual term, representing remaining unexplained variance.

An analysis of the accuracy results was also conducted using a mixed-effects model to confirm the lack of speed-accuracy trade-off. For simplicity, only significant effects are reported except where comparison with previous studies is warranted.

#### Analysis of variance

For the standard ANOVA analysis, a Subjects analysis was conducted on the same mean RT data, aggregated for SPSS, using a 2 (Block-Order) × 2 (Depiction) × 2 (Manipulability) design. Notice that Manipulability is by necessity treated as a categorical variable for this analysis, and that Block is now Block-Order, a between-subjects variable. The goal of analyzing the data with both a mixed-effects analysis and ANOVA was to demonstrate that the results obtained by the mixed-effects analysis were also supported by more-traditional RT analyses.

## RESULTS

### LINEAR MIXED-EFFECTS ANALYSIS: ANALYSIS OF RT

For the mixed-effects regression analysis, *F*-values were derived for each of the effects of interest. The mixed-effects regression analysis results showed a main effect of Block [*F*(1) = 376.98], AoA [*F*(1) = 43.75], Familiarity [*F*(1) = 4.82], Depiction [*F*(1) = 17.67], but not Manipulability [*F*(1) = 0.24]. The effects were in the direction of faster RTs in block 1 compared to block 2, faster RTs for more familiar objects, and those fasters RTs for objects acquired at a younger age (low AoA). In addition, the results showed that RTs were generally faster for photographs of objects as compared to line drawings.

**FIGURE 2:**
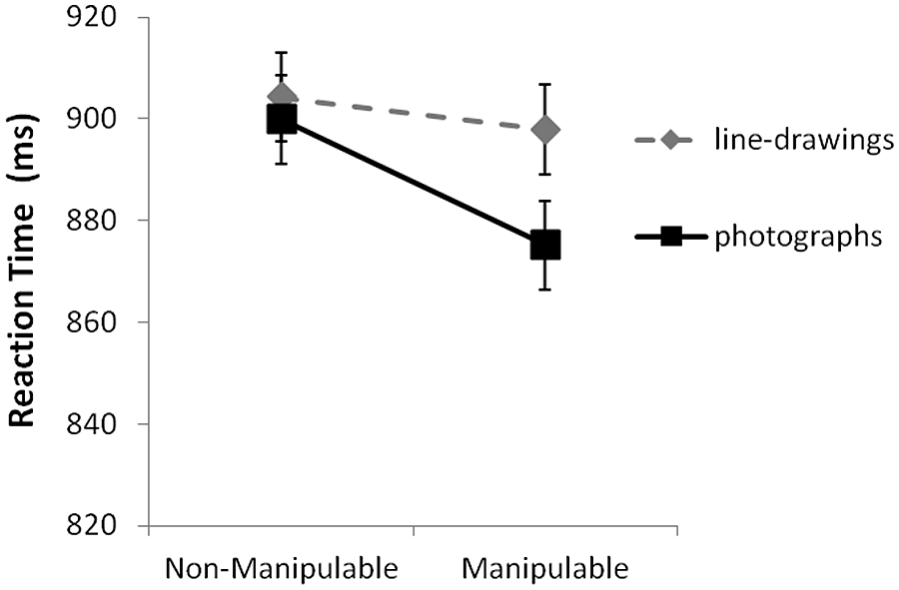
**The reaction time (RT) means for the two-way interaction between Depiction and Manipulability, showing larger effects of object depiction (photographs versus line drawings) for manipulable objects.** Error bars shown indicate confidence intervals calculated according to the method described by Masson and Loftus (2003; see also Loftus and Masson, 1994).

Importantly, however, the results also showed a significant interaction between Manipulability and Depiction [*F*(1) = 9.28], suggesting a bigger Manipulability effect for photographs of objects compared to line-drawings (see **Figure 2** for a depiction of this interaction with Manipulability plotted as a categorical variable for simplicity). Follow-up *post hoc* analyses did not support a significant main effect of Manipulability when just photographs were considered [*F*(1) = 1.23]. However, they did support a significant main effect of object depiction for manipulable objects [*F*(1) = 25.47], but not non-manipulable objects [*F*(1) = 1.09]. These results indicated that manipulable objects were named more quickly as photographs than line drawings, but there was no difference in the time to name non-manipulable objects based on object depiction (line drawings versus photographs). That is, this result supported the notion that manipulable objects are more readily identified when portrayed with more realistic surface detail (such as that in a photograph). Importantly non-manipulable objects did not show this manipulable-object benefit.

#### Linear mixed-effects analysis: analysis of accuracy

A similar mixed effects regression analysis on accuracy (binomial/logit method) was conducted. Results yielded only significant main effects of Block (*z* = 14.04, *p* < 0.001) and AoA (*z* = -3.54, *p* < 0.001). There were no effects of object depiction, manipulability nor interaction between the two, with *z* = -0.11, *p* = 0.913, *z* = -1.68, *p* = 0.093, and *z* = 0.31, *p* = 0.756, respectively. Thus, there was no evidence of a speed accuracy trade-off.

### ANOVA: SUBJECTS ANALYSIS WITH MEAN RTs

To confirm the results obtained with the mixed-effects regression analysis, more standard ANOVAs were conducted. A subjects-analysis was conducted, which required aggregating the same mean RTs from the previous analysis over the 46 participants. The analysis was a 2 (Block-Order) × 2 (Depiction) × 2 (Manipulability) mixed-factors design with Block-Order as a between-subjects variable, and Depiction and Manipulability as within-subjects variables. Note, that for this analysis, Manipulability was now treated as a discrete two-group variable. This was done by classifying all objects with an average Manipulability score of 3.0 or higher as “manipulable” objects, and all other objects as “non-manipulable.” Also note that because this was a subjects-analysis, there was no option to co-vary the effects of “object familiarity” and “AoA” as can easily be done with a mixed-effects analysis. Thus, this ANOVA analysis, by default, had less statistical power than a mixed-effects regression for the following reasons: (1) it could not measure subjects and items variance simultaneously, (2) it could not measure and account for effects of covariates such as object familiarity and AoA, (3) it required treating manipulability as a categorical variable (and not a continuous one).

Despite these limitations, results from the ANOVA indicated a main effect of Manipulability, *F*(1,44) = 7.52, *p* < 0.01, with faster RTs for manipulable as compared to non-manipulable objects (887 ms for manipulable objects and 902 ms for non-manipulable ones). The main effect of Block-Order was not significant, *F*(1,44) = 0.08, *p* = 0.780, and the main effect of Depiction had a trend toward significance, *F*(1,44) = 3.39, *p* = 0.072, with a suggested advantage of faster naming of photographs over line drawings (888 ms for photographs, and 901 ms for line-drawings).

Importantly, the critical interaction between Manipulability and Depiction was significant, *F*(1,44) = 4.49, *p* < 0.05, with the same advantage that was observed in the mixed-effects analysis (see **Figure 2**). Specifically, *post hoc* tests indicated a significant manipulability effect (faster RTs for manipulable objects) for photographs, *F*(1,44) = 18.85, *p* < 0.001, η^2^ = 0.300, but not for line-drawings, *F*(1,44) = 0.57, *p* = 0.453. In addition, *post hoc* tests revealed significant Object Depiction effects (faster RTs for photographs than line-drawings) for manipulable, *F*(1,44) = 9.29, *p* < 0.01, η^2^ = 0.174, but not for non-manipulable objects, *F*(1,44) = 0.20, *p* = 0.653. Therefore, the results from this analysis were consistent with that of the mixed effects analysis showing a larger effect of object depiction for manipulable objects.

The only other significant interaction was the interaction between Depiction and Block-Order, *F*(1,44) = 83.14, *p* < 0.001, which indicated, not surprisingly, that participants were faster at identifying line-drawings when they had already seen the objects as photographs (926 ms for line-drawings shown in block 2, compared to 930 ms if shown in block 1), and faster at identifying photographs when then had already seen them as line-drawings (849 ms for photographs shown in block 2, compared to 872 ms if shown in block 1).

### RESULTS SUMMARY (ACROSS ANALYSES)

A common picture arose from the three analyses described above. First, although a large main effect of manipulability was not supported by this data (i.e., only the subject-analysis supported a main effect), a significant interaction was found between object Depiction and Manipulability. Specifically, these results indicated that for manipulable objects the effect of object depiction was much larger than it was for non-manipulable objects. Manipulable objects presented as photographs were identified significantly more quickly than the same manipulable objects presented as line-drawings. This interaction was supported both by the linear mixed effects regression analysis, and the more traditional ANOVA.

In addition, our results suggested some advantage for identifying photographs faster than line-drawings. Consistent with our findings, previous research (Price and Humphreys, 1989) has supported this advantage for naming black and white photographs over black and white line-drawings.

## DISCUSSION

In this study, photographs and line-drawings of manipulable and non-manipulable objects were presented for speeded naming on a computer screen. We hypothesized that objects with motor associations (i.e., manipulable objects) would be named more quickly when presented as photographs than as line drawings. This hypothesis was based on the idea that more realistic depictions should lead to more motor facilitation and greater activation of embodied representations that would then facilitate naming of such manipulable objects.

In support of this hypothesis, we found a significant interaction between object manipulability and object depiction (photographs versus line-drawings). Manipulable objects, such as “pen” and “potato,” were named more quickly when shown as black and white photographs than when shown as line drawings. In contrast, non-manipulable objects, such as “table” or “bear,” showed no difference in time to name when shown as black and white photographs or line-drawings. In other words, a manipulability effect was found for objects depicted as photographs, but not for the same objects depicted as line-drawings, such as the Snodgrass and Vanderwart (1980) set. This interaction was supported both with a more traditional repeated-measures ANOVA analysis, and a linear mixed-effects analysis. The results were also consistent with our hypothesis that manipulable objects are identified more quickly relative to non-manipulable objects when shown as photographs due to the extra texture and surface detail afforded during identification. These results suggest that additional surface detail and texture in photographs more completely activate embodied representations that are active during object naming.

The present results are important in confirming the manipulability effect; that is, that the property of manipulability confers an advantage on object naming (as observed in Salmon et al., 2014). We extend this finding by showing that this advantage is optimized with increasing visual detail such as detail present in a photograph relative to a line-drawing.

There are a small number of caveats to our interpretations. First, the effect sizes reported here are not large, i.e., *F*(1,44) = 4.49, *p* < 0.05, η^2^ = 0.094, for the interaction in the ANOVA, and *F*(1) = 9.28 for the interaction in the mixed effects regression. This small effect size may explain why other researchers have found weaker effects of surface detail compared to say those of color (e.g., Brodie et al., 1991; Wurm et al., 1993; Tanaka and Presnell, 1999; Rossion and Pourtois, 2004; Laws and Hunter, 2006; Therriault et al., 2009). Certainly, these results suggest that although the link between object depiction (line drawing versus photographs) and manipulability is real, the effect size is small and may depend on both a large sample of items (i.e., 222 objects in the current case), and large sample of participants (i.e., *N* = 46 participants).

Secondly, we have not measured the neural involvement of motor representations in the effects we observe here. One prediction stemming from our behavioral research on the manipulability effect is that the activation of motor areas often observed in neuro-imaging studies during manipulable object naming (e.g., Chao and Martin, 2000) will be larger (or more extensive) for photographs than for line drawings. Future research should investigate this possibility. Of note, some recent research has shown that using real world or 3-D objects can further mediate patterns of results typically observed with pictures of manipulable objects (cf. Snow et al., 2011). However comparisons between manipulable and non-manipulable 3-D *real-world* objects remain elusive because of difficulty of bringing a large sample of real non-manipulable objects into the laboratory.

Despite not measuring neural activity directly, our behavioral results are consistent with the prediction of theory of embodied cognition that photographs should activate more robust embodied object representations than line-drawings, and, to our knowledge, it is the first to provide evidence that the manipulability effect differs for objects that vary only in the details of their depiction.

## CONCLUSION

In conclusion, the results of the current research suggest that the manipulability effect on object naming is sensitive to surface detail and/or object depiction (photographs versus line-drawings). These results indicate that an advantage for identifying manipulable objects is more likely when these objects are presented as photographs than when presented as line-drawings. This result has implications for our understanding of effects of manipulability during object identification tasks, and the factors that moderate such effects. Specifically they reveal that manipulability effects are influenced by the surface details of objects, and suggest that embodied object representations may be more robustly and extensively accessed using more realistic depictions of objects.

## Conflict of Interest Statement

The authors declare that the research was conducted in the absence of any commercial or financial relationships that could be construed as a potential conflict of interest.

## Footnotes

1 In this context we define affordances similarly to Tucker and Ellis (1998). These authors defined affordances as the ways in which objects have the potential to elicit specific motor acts.

## SUPPLEMENTARY MATERIAL

Please see attached supplementary documents that include (1) a table of objects used in this research, and (2) a figure showing all new line-drawings that were created for this research. The Supplementary Material for this article can be found online at: http://www.frontiersin.org/journal/10.3389/fpsyg.2014.01187/abstract
