# Supplementary material for: Photographs of manipulable objects are named more quickly than the same objects depicted as line-drawings: Evidence that photographs engage embodiment more than line-drawings
Source: Front Psychol. 2014 Oct 21;5:1187. doi: 10.3389/fpsyg.2014.01187 (PMC4204636; doi:10.3389/fpsyg.2014.01187)
Supplement: Supplementary file 1 [file Table_1.DOCX]

**Supplementary Table**

List of the 222 objects/items used

| accordion | drum | pepper (bell) |
| --- | --- | --- |
| airplane ^3^ | dryer ^3^ * | piano |
| anchor | duck ^4^ | pigeon ^4^ * |
| ant ^2^ | eagle ^4^ | pineapple^2^ |
| ashtray | egg ^2^ * | pipe |
| axe | elephant | pitcher |
| baby bib ^1^ * | envelope | pliers |
| baby bottle ^1^ | fence | plug |
| baby carriage | fire hydrant ^3^ * | pool table ^3^ * |
| balloon ^1^ | fish ^2^ | pot |
| banana ^2^ | flower ^2^ | potato ^2^ |
| barn ^3^ | fly ^4^ | present ^1^ * |
| barrel | football | rabbit ^2^ |
| basket | forest ^4^ * | raccoon |
| bathtub ^3^ * | fork ^1^ | raspberry ^2^ * |
| bear ^4^ | fox | refrigerator ^3^ |
| bee ^4^ | frog ^2^ | ring |
| beetle ^4^ | frying pan | road ^3^ * |
| bell (church)  ^3^ | garbage can^1^ | roof ^3^ * |
| belt | giraffe ^4^ | rooster ^4^ |
| bicycle ^3^ | glasses ^1^ | ruler ^1^ |
| bird (chickadee)  ^4^ | glove | sailboat ^3^ |
| book | goat | salt shaker ^1^ |
| boot | gorilla | sandwich |
| bottle | grapes ^2^ | saw |
| bow ^1^ | grass ^4^ * | scissors ^1^ |
| bowl | guitar | screw |
| box | gun | screwdriver |
| bread | hammer | seahorse |
| broom | harp | seal |
| brush ^1^ | hat | sheep ^4^ |
| bus | helicopter ^3^ | shell ^2^ * |
| butterfly ^4^ | horse | sink ^3^ * |
| cake ^1^ | house ^3^ | sippy cup ^1^ * |
| camel | iron | skirt |
| candle | ironing board ^1^ | sled ^1^ |
| candy canes ^1^ * | jacket | snail ^2^ |
| cannon | kangaroo | snake ^4^ |
| cap | kettle | sock |
| car ^3^ | key | spider ^2^ |
| carrot ^2^ | kite | stairs ^3^ * |
| cat ^2^ | knife ^1^ | stool ^1^ |
| celery ^2^ | ladder | stove ^3^ |
| chain | lamp ^1^ | strawberry ^2^ |
| chair ^1^ | leaf ^2^ | suitcase |
| chalkboard ^3^ * | lemon ^2^ | sweatshirt |
| cherry ^2^ | leopard | table ^3^ |
| chicken ^4^ | lettuce ^2^ | telephone |
| chisel | light bulb | television ^3^ |
| church ^3^ | lion ^4^ | thread (spool) |
| cigar | lobster ^2^ | tie |
| cigarette | lock ^1^ | tiger |
| clock | mitten ^1^ | toaster ^1^ |
| closet ^3^ * | mixer ^1^ * | toilet ^3^ * |
| clothespin | monkey ^4^ | tomato ^2^ |
| clouds ^4^ | moon ^4^ | toothbrush |
| clown | motorcycle | train |
| comb | mountain ^4^ | tree ^4^ |
| computer ^3^ * | mouse ^2^ | truck ^3^ |
| couch ^3^ | mushroom ^2^ | turtle |
| cow ^4^ | nail | umbrella |
| crab ^4^ * | necklace | vase |
| crocodile | onion ^2^ | vest |
| crown | orange | violin |
| cup | ostrich | wagon |
| deer ^4^ | owl ^4^ | washing machine ^3^ * |
| desk ^3^ | pacifier ^1^ * | watch |
| dog ^4^ | paintbrush ^1^ | well |
| doll | pants | wheels ^1^ |
| donkey | peach ^2^ | whistle |
| door | peacock ^4^ | windmill |
| dragonfly ^4^ * | pear ^2^ | window |
| dress | pen ^1^ | wrench |
| dresser ^3^ | penguin | zebra |
|  |  |  |

1. comes from set of “Manipulable (Manmade)” used by Salmon, Matheson & McMullen, 2014
2. comes from set of “Manipulable (Natural)” used by Salmon, Matheson & McMullen, 2014
3. comes from set of “Non-Manipulable (Manmade)” used by Salmon, Matheson & McMullen, 2014
4. comes from set of “Non-Manipulable (Natural)” used by Salmon, Matheson & McMullen, 2014

* New line drawings created for this comparison (all other line drawings came from Snodgrass and Vanderwart, 1980). For a depiction of the new line drawing artwork created for this experiment, please refer to Supplementary Figure.
