# Supplementary figures and images for: Photographs of manipulable objects are named more quickly than the same objects depicted as line-drawings: Evidence that photographs engage embodiment more than line-drawings
Source: Front Psychol. 2014 Oct 21;5:1187. doi: 10.3389/fpsyg.2014.01187 (PMC4204636; doi:10.3389/fpsyg.2014.01187)

**Supplementary Figure**. New Line Drawings (by Andrea Rankin)

| 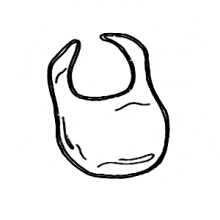 | 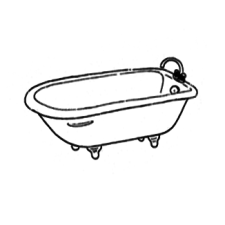 | 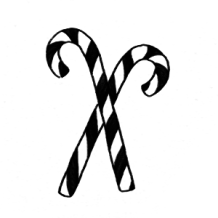 | 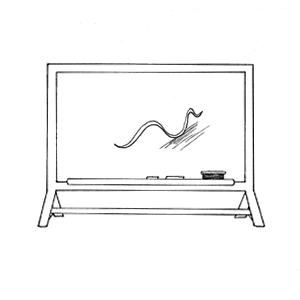 |
| --- | --- | --- | --- |
| 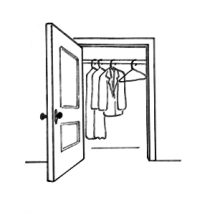 | 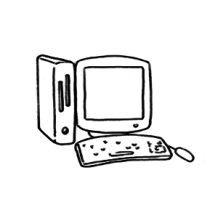 | 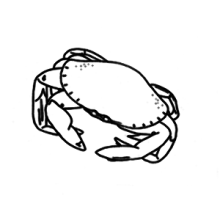 | 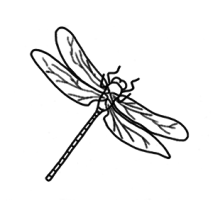 |
| 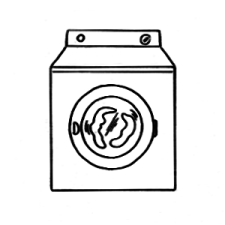 | 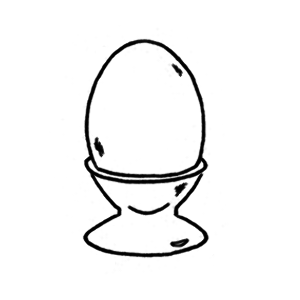 | 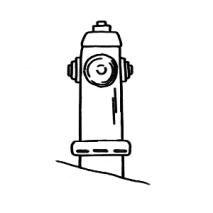 | 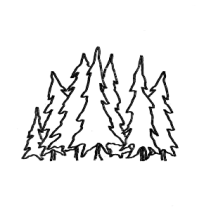 |
| 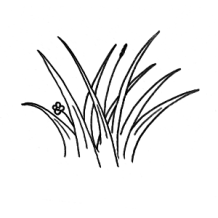 | 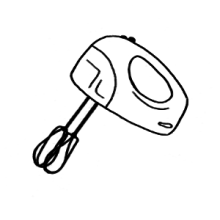 | 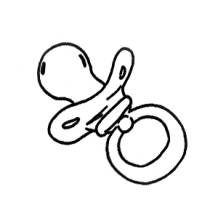 | 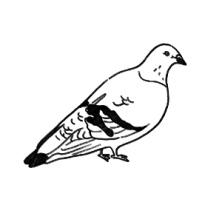 |
| 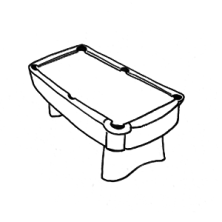 | 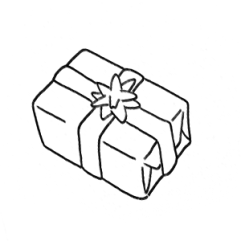 | 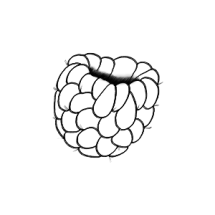 | 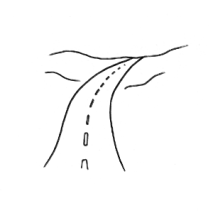 |
| 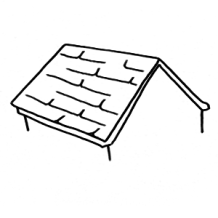 | 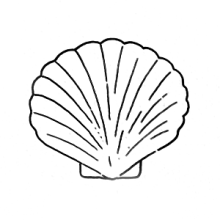 | 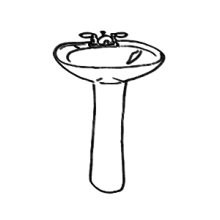 | 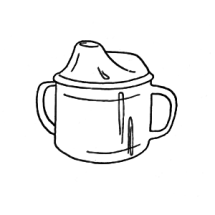 |
| 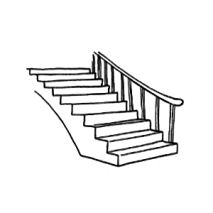 | 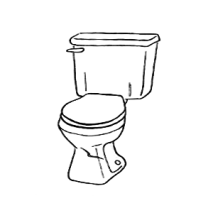 | 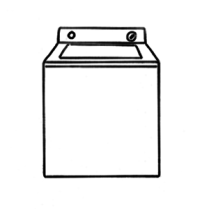 |  |

Supplement: Supplementary file 2 [file Data_Sheet_1.DOCX]
